# A generic cycling hypoxia-derived prognostic gene signature: application to breast cancer profiling

**DOI:** 10.18632/oncotarget.2285

**Published:** 2014-07-31

**Authors:** Romain Boidot, Samuel Branders, Thibault Helleputte, Laila Illan Rubio, Pierre Dupont, Olivier Feron

**Affiliations:** ^1^ Institut de Recherche Expérimentale et Clinique (IREC), Pole of Pharmacology and Therapeutics (FATH), Université catholique de Louvain, Brussels, Belgium; ^2^ Machine Learning Group, Institute of Information and Communication Technologies, Electronics and Applied Mathematics (ICTEAM), Université catholique de Louvain, Louvain-la-Neuve, Belgium

**Keywords:** hypoxia, breast cancer, biomarker, gene signature, prognosis

## Abstract

**Background:**

Temporal and local fluctuations in O_2_ in tumors require adaptive mechanisms to support cancer cell survival and proliferation. The transcriptome associated with cycling hypoxia (CycHyp) could thus represent a prognostic biomarker of cancer progression.

**Methods:**

We exposed 20 tumor cell lines to repeated periods of hypoxia/reoxygenation to determine a transcriptomic CycHyp signature and used clinical data sets from 2,150 breast cancer patients to estimate a prognostic Cox proportional hazard model to assess its prognostic performance.

**Results:**

The CycHyp prognostic potential was validated in patients independently of the receptor status of the tumors. The discriminating capacity of the CycHyp signature was further increased in the ER+ HER2- patient populations including those with a node negative status under treatment (HR=3.16) or not (HR=5.54). The CycHyp prognostic signature outperformed a signature derived from continuous hypoxia and major prognostic metagenes (P<0.001). The CycHyp signature could also identify ER+HER2 node-negative breast cancer patients at high risk based on clinicopathologic criteria but who could have been spared from chemotherapy and inversely those patients classified at low risk based but who presented a negative outcome.

**Conclusions:**

The CycHyp signature is prognostic of breast cancer and offers a unique decision making tool to complement anatomopathologic evaluation.

## INTRODUCTION

Hypoxia is nowadays described as a hallmark of tumors [1, 2]. Tumor angiogenesis and glycolytic metabolism are two extensively studied responses of cancer cells to a deficit in oxygen [1]. The building of new blood vessels to bring O_2_ and the respiration-independent metabolism to survive under low O_2_ are actually complementary responses of tumors to hypoxia [1, 2]. These somehow opposite modes of adaptation account for local and temporal heterogeneities in tumor O_2_ distribution. The terms ‘intermittent hypoxia’ or ‘cycling hypoxia’ were settled to describe this phenomenon of fluctuating hypoxia in tumors [3, 4]. As a corollary, the extent of cycling hypoxia reflects tumor plasticity and thus measures the capacity of tumor cells to survive and proliferate in a hostile environment [3].

Although we and others have contributed to demonstrate the existence of cycles of hypoxia and/or ischemia in mouse, canine and human tumors [see [5, 6] for review], technologies aiming to routinely measure tumor O_2_ fluctuations in the clinics are not (yet) available despite important progresses in the *in vivo* imaging of hypoxia [7-11]. In the absence of readily accessible monitoring strategies, the analysis of the transcriptome associated with this phenomenon could represent a prognostic biomarker of cancer progression. Indeed, although mutations and defects in tumor suppressor genes directly influence the whole genetic profile of a given tumor cell clone, cycling hypoxia could be envisioned as a supra-oncogenic phenomenon influencing gene expression [3]. In other words, independently of the genetic background of tumor cells, cycling hypoxia has the potential to lead to common alterations in the expression of some transcripts, and thus to a possible clinically exploitable signature.

Clinical data sets derived from breast cancer patients could be used to evaluate the performance of such cycling hypoxia-related gene signature. The clinical and genetic heterogeneities of this disease and the very large panel of data sets available represent indeed good opportunities to evaluate new prognostic gene expression signatures [12]. Whole genome analysis already provided several molecular classifications for breast cancer beyond standard clinicopathologic variables [12-21]. The latter include tumor size, presence of lymph node metastasis and histological grades [22] but also encompass three predictive markers of response, namely expression of oestrogen (ER), progesterone (PR) and HER2 receptors [12]. Treatment guidelines are nowadays still largely based on algorithms integrating these informations such as the Notthingham Prognostic Index [22, 23] or Adjuvant! Online [24]. Accordingly, for early-stage breast cancer, adjuvant chemotherapy is recommended for most patients with ER-negative or HER2-positive tumors [13, 25-27]. The challenge actually resides in selecting patients with ER-positive HER2-negative disease who could benefit from chemotherapy.

In this study, we derived a transcriptomic signature of cycling hypoxia (CycHyp) using 20 cell lines derived from various human tumors and characterized by a large variety of distinct genetic anomalies. We then validated the capacity of the CycHyp signature to optimize patient stratification. In particular, we showed how the CycHyp signature could identify ER-positive node-negative breast cancer patients at high risk based on conventional NPI (and who could have been spared from chemotherapy) and inversely those patients classified at low risk but who could have drawn benefits of chemotherapy.

## RESULTS

### Identification of the CycHyp signature

**Figure 1:**
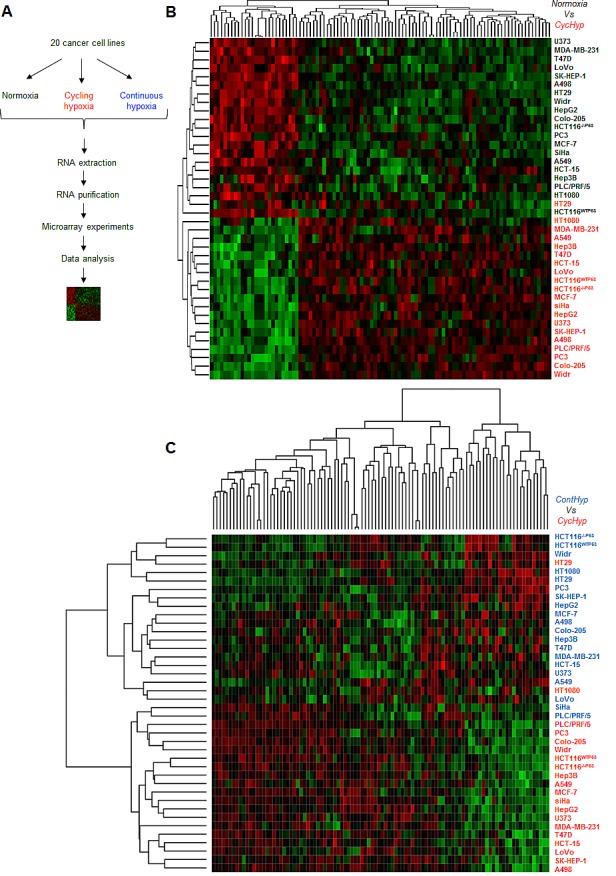
The CycHyp and ContHyp signatures (A.) Flowchart of the signature determination from tumor cells exposed either to normoxia, cycling or continuous hypoxia. (B.) Heatmap depicting the transcripts from the CycHyp signature either underexpressed (green) or overexpressed (red) (centered to median values). Each column corresponds to a specific human Gene 1.0 ST probeset ; each line represents a specific cell line either maintained under normoxia (black label) or exposed to cycling hypoxia (red label); cells under normoxia and cycling hypoxia are perfectly separated in two distinct clusters, except for one cycling hypoxia sample in the normoxia cluster. (C.) Similarly, a heatmap depicting the relative expression of transcripts from the CycHyp signature in the cell lines maintained under continuous hypoxia (blue) or cycling hypoxia (red); only two cycling hypoxia samples are grouped with the continuous hypoxia samples.

Tumor cells covering a large diversity of tissues (Suppl. Table 1) were submitted to cycling hypoxia (CycHyp) for 24 hours, maintained under normoxic conditions or exposed to continuous hypoxia (ContHyp) for the same period of time (Figure 1A). Corresponding mRNA samples were analysed by hybridization using Human Gene 1.0 ST Affymetrix microarrays. Gene expression profiles of each cell type under normoxia *vs.* cycling hypoxia (CycHyp) were produced to identify the most differentially expressed probesets. The CycHyp signature was determined as the top 100 probesets with the lowest FDR-corrected p-values averaged over 200 resamplings (Table 1); a ContHyp signature was also determined in parallel (Table 2). The heatmaps made with the 100 probe sets of the CycHyp signature confirmed its excellent potential of discrimination between cycling hypoxia and either normoxia (Figure 1B) or continuous hypoxia (Figure 1C). Moreover, Gene Set Enrichment Analysis (GSEA) [28] indicated that when considering differentially expressed probesets (after FDR correction), only 2 gene sets were significantly enriched in the CycHyp signature (Suppl. Table 2) whereas we identified 52 gene sets enriched in the ContHyp signature, including 17 directly related to hypoxia (Suppl. Table 3). Also, when using the MSigDB molecular signature database referring to hypoxia or HIF (www.broadinstitute.org), we found 13 hypoxia gene sets sharing, on average, only 1.4 gene with CycHyp (Suppl. Table 4) whereas 44 hypoxia gene sets showed overlap with ContHyp with an average of 6.6 (1-27) common genes (Suppl. Table 5). We also compared the CycHyp signature to 13 other hypoxia-derived signatures described by Seigneuric et al. [29] and Starmans et al. [30]. The CycHyp signature was again far from those signatures with an average of only 1 gene in common. The overlap was larger between ContHyp and those signatures with an average of 6 genes in common (Suppl. Table 6). Finally, using TFactS [31] to analyse transcription factors regulating expression of genes associated to either signature, HIF-1α was only found as positively associated with the ContHyp signature.

**Table 1:** Gene list of the CycHyp signature

	Probe	Entrez ID	GenBank	Symbol	Gene Title
1	8018860	332	NM_001168	BIRC5	baculoviral IAP repeat containing 5
2	8064156	84619	NM_032527	ZGPAT ^*^	zinc finger, CCCH-type with G patch domain
3	8138912	23658	NM_012322	LSM5^§^	LSM5 homolog, U6 small nuclear RNA associated (S. cerevisiae)
4	7921786	5202	NM_012394	PFDN2	prefoldin subunit 2
5	8165011	2219	NM_002003	FCN1	ficolin (collagen/fibrinogen domain containing) 1
6	7964262	4666	NM_001113201	NACA^*^	nascent polypeptide-associated complex alpha subunit
7	7949792	5790	NM_005608	PTPRCAP ^#^	protein tyrosine phosphatase, receptor type, C-associated protein
8	8034101	11018	NM_006858	TMED1	transmembrane emp24 protein transport domain containing 1
9	8168087	3476	NM_001551	IGBP1	immunoglobulin (CD79A) binding protein 1
10	7963575	1975	NM_001417	EIF4B§	eukaryotic translation initiation factor 4B
11	8124397	3006	NM_005319	HIST1H1C ^#^	histone cluster 1, H1c
12	7975989	81892	NM_031210	SLIRP^§^	SRA stem-loop interacting RNA binding protein
13	8127692	3351	NM_000863	HTR1B	5-hydroxytryptamine (serotonin) receptor 1B
14	8127087	2940	NM_000847	GSTA3	glutathione S-transferase alpha 3
15	7941122	29901	NM_013299	SAC3D1	SAC3 domain containing 1
16	7998692	4913	NM_002528	NTHL1	nth endonuclease III-like 1 (E. coli)
17	8073623	758	NM_001044370	MPPED1	metallophosphoesterase domain containing 1
18	8014865	4761	NM_006160	NEUROD2 ^*^	neurogenic differentiation 2
19	8005726	3768	NM_021012	KCNJ12	potassium inwardly-rectifying channel, subfamily J, member 12
20	7966631	64211	NM_022363	LHX5 ^*^	LIM homeobox 5
21	8037853	54958	NM_017854	TMEM160	transmembrane protein 160
22	8104136	3166	NM_018942	HMX1^*^	H6 family homeobox 1
23	7948606	746	NM_014206	C11orf10 ^#^	chromosome 11 open reading frame 10
24	8044773	8685	NM_006770	MARCO	macrophage receptor with collagenous structure
25	7947015	7251	NM_006292	TSG101	tumor susceptibility gene 101
26	7931553	8433	NM_003577	UTF1 ^*^	undifferentiated embryonic cell transcription factor 1
27	7956876	84298	NM_032338	LLPH	LLP homolog, long-term synaptic facilitation (Aplysia)
28	8117372	8334	NM_003512	HIST1H2AC#	histone cluster 1, H2ac
29	8001329	869	NM_004352	CBLN1	cerebellin 1 precursor
30	8027205	51079	NM_015965	NDUFA13	NADH dehydrogenase (ubiquinone) 1 alpha subcomplex, 13
31	8042896	3196	NM_016170	TLX2 ^*^	T-cell leukemia homeobox 2
32	7911532	54998	NM_017900	AURKAIP1	aurora kinase A interacting protein 1
33	8039923	54998	NM_017900	AURKAIP1	aurora kinase A interacting protein 1
34	7992043	65990	BC001181	FAM173A	family with sequence similarity 173, member A
35	8063074	90204	NM_080603	ZSWIM1 ^*^	zinc finger, SWIM-type containing 1
36	7992191	23430	NM_012217	TPSD1	tryptase delta 1
37	8108435	7322	NM_181838	UBE2D2	ubiquitin-conjugating enzyme E2D 2
38	8165309	8721	NM_003792	EDF1 ^*^	endothelial differentiation-related factor 1
39	7946267	63875	NM_022061	MRPL17	mitochondrial ribosomal protein L17
40	7945536	51286	NM_016564	CEND1	cell cycle exit and neuronal differentiation 1
41	8159609	8636	NM_003731	SSNA1 ^#^	Sjogren syndrome nuclear autoantigen 1
42	8005471	6234	NM_001031	RPS28 ^#^,^§^	ribosomal protein S28
43	8025395	6234	NM_001031	RPS28	ribosomal protein S28
44	7942824	6234	NM_001031	RPS28	ribosomal protein S28
45	8170753	26576	NM_014370	SRPK3	SRSF protein kinase 3
46	8032718	1613	NM_001348		
47	7967067	8655	NM_001037495		
48	8159654	25920	NM_015456	COBRA1 ^*^	cofactor of BRCA1
49	8011212	6391	NM_003001	SDHC	succinate dehydrogenase complex, subunit C, integral membrane protein, 15kDa
50	8011968	51003	NM_016060	MED31 ^*^	mediator complex subunit 31
51	7977440	9834	NR_026800	KIAA0125	KIAA0125
52	8016508	11267	NM_007241	SNF8 ^*^	SNF8, ESCRT-II complex subunit, homolog (S. cerevisiae)
53	8168567	5456	NM_000307	POU3F4 ^*^	POU class 3 homeobox 4
54	8086317	64689	NM_031899	GORASP1	golgi reassembly stacking protein 1, 65kDa
55	8052834	54980	BC005079	C2orf42	chromosome 2 open reading frame 42
56	8073334	9978	NM_014248	RBX1 ^#^	ring-box 1, E3 ubiquitin protein ligase
57	7915846	8569	NM_003684	MKNK1	MAP kinase interacting serine/threonine kinase 1
58	8071920	6634	NM_004175	SNRPD3 ^§^	small nuclear ribonucleoprotein D3 polypeptide 18kDa
59	8032371	81926	NM_031213	FAM108A1	family with sequence similarity 108, member A1
60	7924884	8290	NM_003493	HIST3H3	histone cluster 3, H3
61	8006845	6143	NM_000981	RPL19 ^§^	ribosomal protein L19
62	7946812	6207	NM_001017	RPS13 ^#^,^§^	ribosomal protein S13
63	7949015	65998	NM_001144936	C11orf95 ^*^	chromosome 11 open reading frame 95
64	8009784	51081	NM_015971	MRPS7 ^§^	mitochondrial ribosomal protein S7
65	8174509	2787	NM_005274	GNG5	guanine nucleotide binding protein (G protein), gamma 5
66	7906235	5546	NM_005973	PRCC ^§^	papillary renal cell carcinoma (translocation-associated)
67	8020179	57132	NM_020412	CHMP1B	chromatin modifying protein 1B
68	7947450	4005	NM_005574	LMO2	LIM domain only 2 (rhombotin-like 1)
69	8064370	6939	NM_004609	TCF15 ^*^	transcription factor 15 (basic helix-loop-helix)
70	7955896	22818	NM_016057	COPZ1	coatomer protein complex, subunit zeta 1
71	8137805	8379	NM_003550	MAD1L1 ^#^	MAD1 mitotic arrest deficient-like 1 (yeast)
72	8117334	8359	NM_003538	HIST1H4A ^#^	histone cluster 1, H4a
73	8117368	8364	NM_003542	HIST1H4C ^#^	histone cluster 1, H4c
74	7977507	85495	NR_002312	RPPH1^§^	ribonuclease P RNA component H1
75	7949410	378938	BC018448	MALAT1	metastasis associated lung adenocarcinoma transcript 1 (non-protein coding)
76	8150433	157848	NM_152568	NKX6-3 ^*^	NK6 homeobox 3
77	8071168	29797	NR_024583	POM121L8P	POM121 membrane glycoprotein-like 8 pseudogene
78	7989611	84191	NM_032231	FAM96A	family with sequence similarity 96, member A
79	7980859		NM_001080113		
80	8032782	126259	NM_144615	TMIGD2	transmembrane and immunoglobulin domain containing 2
81	8110861	64979	NM_032479	MRPL36 ^§^	mitochondrial ribosomal protein L36
82	7901687	199964	NM_182532	TMEM61	transmembrane protein 61
83	7916130	112970	NM_138417	KTI12	KTI12 homolog, chromatin associated (S. cerevisiae)
84	8048712	440934	BC033986	LOC440934	hypothetical LOC440934
85	8018993	146713	NM_001082575	RBFOX3 ^§^	RNA binding protein, fox-1 homolog (C. elegans) 3
86	8032601	84839	NM_032753	RAX2	retina and anterior neural fold homeobox 2
87	8010719	201255	NM_144999	LRRC45	leucine rich repeat containing 45
88	8036584	3963	NM_002307	LGALS7	lectin, galactoside-binding, soluble, 7
89	8133209	441251	NR_003666	SPDYE7P	speedy homolog E7 (Xenopus laevis), pseudogene
90	8159501	286256	NM_178536	LCN12	lipocalin 12
91	8028546	3963	NM_002307	LGALS7	lectin, galactoside-binding, soluble, 7
92	8065013		ENST00000427835		
93	8018502	201292	NM_173547	TRIM65 ^*^	tripartite motif containing 65
94	7903294	64645	NM_033055	HIAT1	hippocampus abundant transcript 1
95	7989473	388125	NM_001007595	C2CD4B	C2 calcium-dependent domain containing 4B
96	8054449	644903	AK095987	FLJ38668	hypothetical LOC644903
97	8081867	51300	NM_016589	TIMMDC1	translocase of inner mitochondrial membrane domain containing 1
98	7934544	118881	NM_144589	COMTD1	catechol-O-methyltransferase domain containing 1
99	7968260	219409	NM_145657	GSX1 ^*^	GS homeobox 1
100	8022952	56853	NM_020180	CELF4 ^§^	CUGBP, Elav-like family member 4

^#^ common to the ContHyp signature

^*^ regulators of transcription

^§^ involved in RNA processing

**Table 2:** Gene list of the ContHyp signature

	Probe	Entrez ID	GenBank	Symbol	Gene Title
1	7948606	746	NM_014206	C11orf10	chromosome 11 open reading frame 10
2	8043283	55818	NM_018433	KDM3A	lysine (K)-specific demethylase 3A
3	8025395	6234	NM_001031	RPS28	ribosomal protein S28
4	8139706	23480	NM_014302	SEC61G	Sec61 gamma subunit
5	7942824	6234	NM_001031	RPS28	ribosomal protein S28
6	8005471	6234	NM_001031	RPS28	ribosomal protein S28
7	8048489	55139	NM_018089	ANKZF1	ankyrin repeat and zinc finger domain containing 1
8	7994737	226	NM_000034	ALDOA	aldolase A, fructose-bisphosphate
9	7934278	5033	NM_000917	P4HA1	prolyl 4-hydroxylase, alpha polypeptide I
10	8102518	401152	NM_001170330	C4orf3	chromosome 4 open reading frame 3
11	8117334	8359	NM_003538	HIST1H4A	histone cluster 1, H4a
12	8074969	1652	NM_001355	DDT	D-dopachrome tautomerase
13	8044766	51141	NM_016133	INSIG2	insulin induced gene 2
14	7937476	6181	NM_001004	RPLP2	ribosomal protein, large, P2
15	8086961	5210	NM_004567	PFKFB4	6-phosphofructo-2-kinase/fructose-2,6-biphosphatase 4
16	8145454	665	NM_004331	BNIP3L	BCL2/adenovirus E1B 19kDa interacting protein 3-like
17	8113981	8974	NM_004199	P4HA2	prolyl 4-hydroxylase, alpha polypeptide II
18	8162142	81689	NM_030940	ISCA1	iron-sulfur cluster assembly 1 homolog (S. cerevisiae)
19	8007992	3837	NM_002265	KPNB1	karyopherin (importin) beta 1
20	7928308	54541	NM_019058	DDIT4	DNA-damage-inducible transcript 4
21	8073334	9978	NM_014248	RBX1	ring-box 1, E3 ubiquitin protein ligase
22	8124397	3006	NM_005319	HIST1H1C	histone cluster 1, H1c
23	8153459	65263	NM_023078	PYCRL	pyrroline-5-carboxylate reductase-like
24	7916568		AF263547		
25	7955117	23519	NM_012404	ANP32D	acidic (leucine-rich) nuclear phosphoprotein 32 family, member D
26	8098604	353322	NM_181726	ANKRD37	ankyrin repeat domain 37
27	8121076	10957	NM_006813	PNRC1	proline-rich nuclear receptor coactivator 1
28	7921076	54865	NM_182679	GPATCH4	G patch domain containing 4
29	7908879	8497	NM_015053	PPFIA4	protein tyrosine phosphatase, receptor type, f polypeptide (PTPRF), interacting protein (liprin), alpha 4
30	8103518	23520	NM_012403	ANP32C	acidic (leucine-rich) nuclear phosphoprotein 32 family, member C
31	8050591	91942	NM_174889	NDUFAF2	NADH dehydrogenase (ubiquinone) 1 alpha subcomplex, assembly factor 2
32	8172154	6187	NM_002952	RPS2	ribosomal protein S2
33	7984846	1198	NM_001130028	CLK3	CDC-like kinase 3
34	7946812	6207	NM_001017	RPS13	ribosomal protein S13
35	7982531	8125	NM_006305	ANP32A	acidic (leucine-rich) nuclear phosphoprotein 32 family, member A
36	8119898	7422	NM_001025366	VEGFA	vascular endothelial growth factor A
37	8004331	9744	NM_014716	ACAP1	ArfGAP with coiled-coil, ankyrin repeat and PH domains 1
38	8159441	29085	NM_001135861	PHPT1	phosphohistidine phosphatase 1
39	8168500	5230	NM_000291	PGK1	phosphoglycerate kinase 1
40	7938890	10196	NM_005788	PRMT3	protein arginine methyltransferase 3
41	7930398	4601	NM_005962	MXI1	MAX interactor 1
42	7997740	81631	NM_022818	MAP1LC3B	microtubule-associated protein 1 light chain 3 beta
43	8004360	147040	NM_001002914	KCTD11	potassium channel tetramerisation domain containing 11
44	7909782	51018	NM_016052	RRP15	ribosomal RNA processing 15 homolog (S. cerevisiae)
45	7949792	5790	NM_005608	PTPRCAP	protein tyrosine phosphatase, receptor type, C-associated protein
46	8124385	8366	NM_003544	HIST1H4B	histone cluster 1, H4b
47	8117368	8364	NM_003542	HIST1H4C	histone cluster 1, H4c
48	8081241	84319	NM_032359	C3orf26	chromosome 3 open reading frame 26
49	8050079	246243	NM_002936	RNASEH1	ribonuclease H1
50	8005765	26118	NM_015626	WSB1	WD repeat and SOCS box containing 1
51	7924491	64853	NM_022831	AIDA	axin interactor, dorsalization associated
52	8133273		ENST00000455206		
53	8124391	8335	NM_003513	HIST1H2AB	histone cluster 1, H2ab
54	8159609	8636	NM_003731	SSNA1	Sjogren syndrome nuclear autoantigen 1
55	7957890	27340	NM_014503	UTP20	UTP20, small subunit (SSU) processome component, homolog (yeast)
56	7933582	100287932	NM_006327	TIMM23	translocase of inner mitochondrial membrane 23 homolog (yeast)
57	8153002	10397	NM_001135242	NDRG1	N-myc downstream regulated 1
58	7926037	5209	NM_004566	PFKFB3	6-phosphofructo-2-kinase/fructose-2,6-biphosphatase 3
59	8082066	26355	NM_014367	FAM162A	family with sequence similarity 162, member A
60	8042962	9801	NM_014763	MRPL19	mitochondrial ribosomal protein L19
61	8090678	11222	NM_007208	MRPL3	mitochondrial ribosomal protein L3
62	7977507	85495	NR_002312	RPPH1	ribonuclease P RNA component H1
63	8007397	10197	NM_176863	PSME3	proteasome (prosome, macropain) activator subunit 3 (PA28 gamma/ Ki)
64	7998902	54985	NM_017885	HCFC1R1	host cell factor C1 regulator 1 (XPO1 dependent)
65	8117372	8334	NM_003512	HIST1H2AC	histone cluster 1, H2ac
66	7997230	5713	NM_002811	PSMD7	proteasome (prosome, macropain) 26S subunit, non-ATPase, 7
67	7915485	10969	NM_006824	EBNA1BP2	EBNA1 binding protein 2
68	8113873	3094	NM_005340	HINT1	histidine triad nucleotide binding protein 1
69	7958152	5223	NM_002629	PGAM1	phosphoglycerate mutase 1 (brain)
70	7947867	5702	NM_002804	PSMC3	proteasome (prosome, macropain) 26S subunit, ATPase, 3
71	7964460	1649	NM_004083	DDIT3	DNA-damage-inducible transcript 3
72	7928395	170384	NM_173540	FUT11	fucosyltransferase 11 (alpha (1,3) fucosyltransferase)
73	8163629	944	NM_001244	TNFSF8	tumor necrosis factor (ligand) superfamily, member 8
74	7965486	51134	NM_016122	CCDC41	coiled-coil domain containing 41
75	8136179	23008	AF277175	KLHDC10	kelch domain containing 10
76	8095870	901	NM_004354	CCNG2	cyclin G2
77	8127526	6170	NM_001000	RPL39	ribosomal protein L39
78	8174710	6170	NM_001000	RPL39	ribosomal protein L39
79	8137517	3361	NM_024012	HTR5A	5-hydroxytryptamine (serotonin) receptor 5A
80	7929624	5223	NM_002629	PGAM1	phosphoglycerate mutase 1 (brain)
81	8052331	87178	NM_033109	PNPT1	polyribonucleotide nucleotidyltransferase 1
82	8015969	7343	NM_014233	UBTF	upstream binding transcription factor, RNA polymerase I
83	8069168	386685	NM_198699	KRTAP10-12	keratin associated protein 10-12
84	7941087	5526	NM_006244	PPP2R5B	protein phosphatase 2, regulatory subunit B', beta
85	8026875	26780	NR_000012	SNORA68	small nucleolar RNA, H/ACA box 68
86	8027621	2821	NM_000175	GPI	glucose-6-phosphate isomerase
87	8130539	117289	NM_054114	TAGAP	T-cell activation RhoGTPase activating protein
88	8004691	92162	NM_203411	TMEM88	transmembrane protein 88
89	7962183	205	NM_001005353	AK4	adenylate kinase 4
90	8137805	8379	NM_003550	MAD1L1	MAD1 mitotic arrest deficient-like 1 (yeast)
91	8124388	8358	NM_003537	HIST1H3B	histone cluster 1, H3b
92	8083223	205428	NM_173552	C3orf58	chromosome 3 open reading frame 58
93	8113305	1105	NM_001270	CHD1	chromodomain helicase DNA binding protein 1
94	8169659	4694	NM_004541	NDUFA1	NADH dehydrogenase (ubiquinone) 1 alpha subcomplex, 1, 7.5kDa
95	8046408	5163	NM_002610	PDK1	pyruvate dehydrogenase kinase, isozyme 1
96	8053599	23559	NM_012477	WBP1	WW domain binding protein 1
97	8043377	23559	NM_012477	WBP1	WW domain binding protein 1
98	7960878	642559	GU480887	POU5F1P3	POU class 5 homeobox 1 pseudogene 3
99	7959023	643246	NM_001085481	MAP1LC3B2	microtubule-associated protein 1 light chain 3 beta 2
100	8073148	468	NM_001675	ATF4	activating transcription factor 4 (tax-responsive enhancer element B67)

### The CycHyp signature predicts clinical outcome in breast cancer patients

To evaluate the prognostic value of the CycHyp signature, we focused on breast cancer because of the very large amounts of well-annotated clinical data sets available and a clearly identified need to discriminate between patients at low and high risks among subgroups determined on the basis of clinicopathologic criteria [12, 13]. Publicly available GEO data sets allowed us to collect information on the survival of 2,150 patients with primary breast cancer (see clinical features in Table 3).

**Table 3:** Breast Cancer Patient Demographics and Characteristics

	All patients n = 2150 No %	ER+/HER2- n=1452 No %	ER+/HER2- Node neg. n=899 No %	ER+/HER2- Node neg. Untreated n=590 No %
Age				
≤50 >50 NA	649 30 945 44 556 26	388 27 649 45 415 28	218 24 367 41 314 35	190 32 237 40 163 28
Tumor size				
≤2cm >2cm NA	742 35 473 22 935 43	537 37 326 22 589 41	474 53 210 23 215 24	424 72 158 28 8 1
Grade				
0-1 2 3 NA	224 10 605 28 487 23 834 39	200 14 485 33 206 14 561 39	148 17 346 38 162 18 243 27	104 18 270 46 137 23 79 13
Node status				
Negative Positive	1329 62 821 38	899 62 553 38	899 100 0 0	590 100 0 0
Estrogen receptor				
Negative Positive NA	443 21 1607 75 100 4	0 0 1452 100 0 0	0 0 899 100 0 0	0 0 590 100 0 0
HER2 status				
Negative Positive	1835 85 315 15	1452 100 0 0	899 100 0 0	590 100 0 0
Treatment				
None Chemotherapy Hormonotherapy	901 42 691 32 558 26	590 41 410 28 452 31	590 66 73 8 236 26	590 100 0 0 0 0

Data obtained from GSE11121 (n=200), GSE17705 (n=298), GSE2034/5327 (n=344), GSE20685 (n=327), GSE21653 (n=253), GSE2990 (n=138), GSE3494 (n=178), GSE6532 (n=214), and GSE7390 (n=198). NA = Not Available.

**Figure 2:**
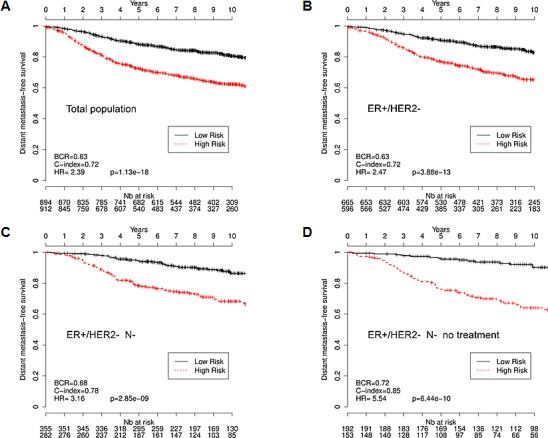
Kaplan-Meier survival curves of patients with primary breast cancer, as determined by using the CycHyp signature (A) All patients. (B.) ER+/HER2- patients, (C.) node-negative ER+/HER2-, (D.) node-negative, untreated ER+/HER2- patients (DFS Mantel-Cox comparison); hazard ratio (HR), balanced classification rate (BCR) and concordance index (C-index) for the prediction in high risk *vs.* low risk groups are reported; HRs are presented with their associated p-values.

In order to exploit these data sets, we first transferred the Gene 1.0ST datasets in the HU133 platform. We then used the VDX dataset (GSE2034 and GSE5327) as a reference because of its large number of node negative untreated patients [17]. This training dataset was used to estimate a prognostic multivariate Cox proportional hazard model built on the CycHyp signature (see Methods for details). The other eight datasets (see references in Table 3) were used according to the methodology described by Haibe-Kains and colleagues [32], to assess the prognostic performance of the CycHyp signature on independent samples. We first chose to evaluate our signature independently of the clinicopathological data. The prognostic potential of the CycHyp signature to discriminate between patients at low or high risk was confirmed with a HR=2.39 and a p-value = 1.13e-18 whathever the treatment and the tumor histology (Figure 2A). We then focused on the ER+ HER2- population which is known to be heterogeneous and thus difficult to treat [12, 13]. The discriminating capacity of the CycHyp signature remained strikingly high in the ER+ HER2- patient populations (HR = 2.47, p-value = 3.88e-13, Figure 2B). Finally, among this subpopulation of patients, we considered those with a node negative status (Figure 2C) and among the latter, those who did not receive any treatment (Figure 2D). Hazard ratios rose to 3.16 and 5.54 in these conditions (p-values = 2.85e-9 and 6.44e-10, respectively), further supporting the discriminating potential of the CycHyp signature. In particular, the data presented in Figure 2D allowed to exclude any confounding influence of the potential benefit arising from the treatment administered to these patients and thus clearly identified a population of patients who remained inadequately untreated.

Using the same methodology, we examined the prognostic capacity of the ContHyp signature (discriminating between normoxia and continuous hypoxia). The performance of the ContHyp signature was satisfactory on the ER+ HER2- untreated population (HR = 2.58, p-value = 1.46e-4, see Supplementary Fig. 1) but was significantly lower (p-value = 3.61e-8) than the CycHyp signature.

### The CycHyp signature provides significant additional prognostic information to available multigene assays

**Figure 3:**
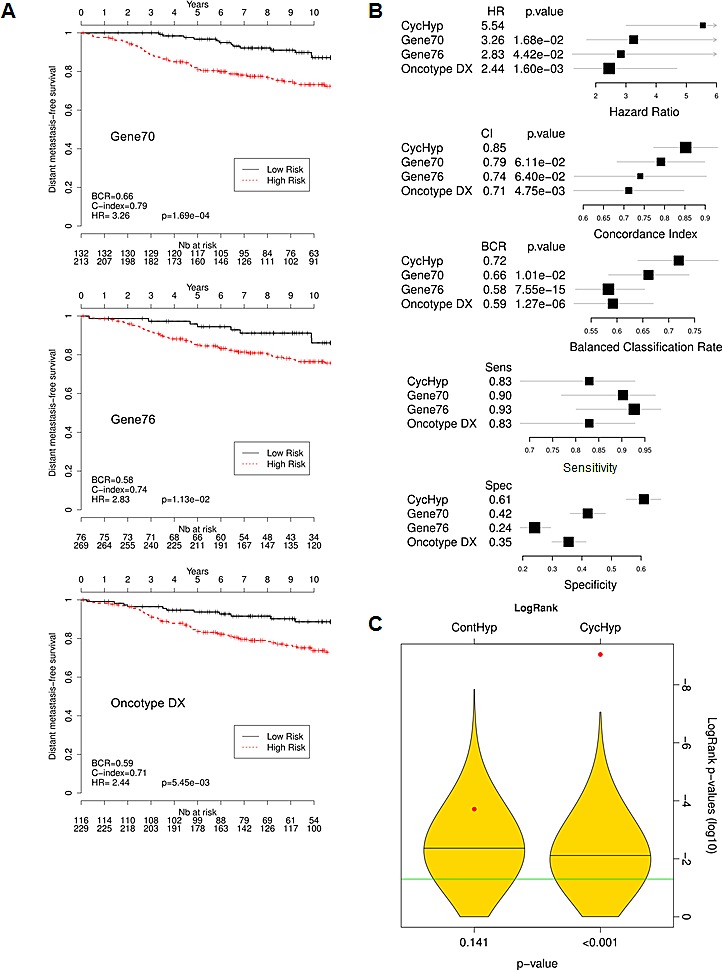
Comparison of the prognostic potential of the CycHyp signature vs. Gene 70 (Mammaprint), Gene 76 and Oncotype Dx signatures (A) Kaplan-Meier survival curves of node-negative, untreated ER+/HER2- patients, as determined by using the indicated signature (DFS Mantel-Cox comparison); hazard ratio (HR), balanced classification rate (BCR) and C-index for the prediction in high risk *vs.* low risk groups are reported; HR are presented with their associated p-values. (B.) Forest plots of the hazard ratio (HR), Concordance index (CI), balance classification rate (BCR), sensitivity and specificity for the prediction in high risk *vs.* low risk groups; p-values refer to the comparisons of CycHyp *vs.* Gene 70 (Mammaprint), Gene 76 and Oncotype Dx. (C.) Graph represents the power of discrimination in high *vs.* low risk groups (expressed as the logarithm of the p-values of the logrank) of the ContHyp and CycHyp signatures (see red dots) versus 1,000 randomly generated signatures (yellow shapes depicting their distribution).

To evaluate the performance of the CycHyp signature, we compared it with other well-established prognostic multigene assays for breast cancer, namely Gene70 or Mammaprint [14], Gene76 [17] and Oncotype Dx [15]. Using the same set of ER+ HER2- node negative patients as used in Figure 2D, we could determine the low *vs.* high risk patient stratification according to these signatures. The superior prognostic potential of the CycHyp signature could be captured from the Kaplan Meier curves obtained with the Gene 70, Gene76 and Oncotype DX signatures (compare Figure 3A with Figure 2D). Hazard ratios confirmed the net advantage of the CycHyp signature with a significantly higher value than the three other metagenes (Figure 3B). The concordance index, which is the probability of a high risk patient to relapse before a low risk patient, was also higher with the CycHyp signature (Figure 3B). Finally, the Balanced Classification Rate (BCR), which represents the average between sensitivity and specificity to discriminate between patients with progressing disease *vs.* disease-free at 5 years, was significantly higher for the CycHyp signature than the three other multigene assays (Figure 3B). The sensitivity of the CycHyp was above 80% and the specificity of the CycHyp signature was well above the level of the others (Figure 3B). Of note, the metrics corresponding to each data set taken separately is depicted in Suppl. Figure 2.

Importantly, to further validate the prognostic significance of the CycHyp signature, a comparison with random gene signatures was performed according to the methodology described by Venet et al. [33] and Beck et al. [34]. Figure 3C shows the distribution of the p-values (logrank test in log 10) for 1000 randomly generated signatures together with the p-values of the CycHyp and ContHyp signatures. The logrank test (or Mantel-Haenszel test) [35] is commonly used to assess whether there is a significant survival difference between risk groups. The discrimination between risk groups was significantly higher (P < 0.001) with the CycHyp signature as compared to each of the random signatures whereas the ContHyp signature was not significantly better (vs. random ones; P=0.141). The same analysis was carried out for the three other metrics (HR, CI and BCR) to assess the discrimination capability between risk groups and confirmed the significantly higher value of the CycHyp signature (vs. random signatures) (Suppl. Figure 3).

### The CycHyp signature in association with NPI offers a powerful prognostic tool

**Figure 4:**
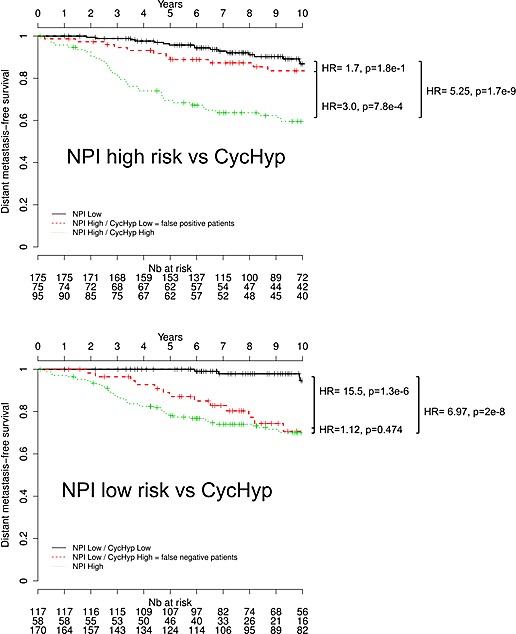
Kaplan-Meier survival curves of node-negative, untreated ER+/HER2- patients stratified by using the CycHyp signature to detect (A.) false positive patients among those identified at high risk based on the NPI nomenclature and (B.) false negative patients among those identified at low risk based on the NPI nomenclature (DFS Mantel-Cox comparison).

We then aimed to determine whether the CycHyp signature could improve the Nottingham Prognostic Index (NPI) for better predicting the survival of operable breast cancers. The NPI algorithm combines nodal status, tumour size and histological grade and allows to model a *continuum* of clinical aggressiveness with 3 subsets of patients divided into good, moderate, and poor prognostic groups with 15-year survival [22, 23, 36]. Since few patients were assigned a poor index, we merged here the moderate and poor indices into a high risk group to facilitate the comparison with the CycHyp signature. We found that by integrating the CycHyp signature, an important proportion of patients could be reclassified to another risk group (Figure 4). 44.1% of patients classified at high risk using the NPI algorithm were identified at low risk when using the CycHyp signature and were confirmed to be “false positive” since they actually exhibited a profile of survival closer to the low risk NPI patient (Figure 4A). Inversely, using the CycHyp signature, we also identified in the patients at low risk based on the NPI criteria, 33.1% of patients with a risk profile closer to the patients with a negative outcome (Figure 4B). This increased discriminating potential remained highly relevant when considering all patients or patients with a ER+ HER2- status (and among the latter, those with a node negative status or the untreated ones) (see Suppl. Figure 4).

## DISCUSSION

This study demonstrates that a gene signature derived from the transcriptomic adaptation of tumor cells to cycling hypoxia is prognostic of breast cancer. The CycHyp signature that we have identified and validated in this study has not only prognostic value independently of molecular risk factors but also provides significant additional prognostic information to clinicopathologic criteria. Clinical outcome of breast cancer patients is nowadays largely based on histological grade and the status of ER, PR, and HER2 receptors [12, 13, 22]. In early breast cancer, a lack of expression of ER (and PR) will almost systematically lead to the administration of adjuvant chemotherapy in addition to locoregional treatment [12, 25, 26]. Also, for patients with a tumor expressing HER2, chemotherapy and/or trastuzumab represents the option the most likely to be beneficial based on current clinical knowledge [12]. The impact of chemotherapy is actually more difficult to anticipate for the rest of early-stage breast cancer patients, i.e. those diagnosed with a ER-positive and HER2-negative disease. These patients represent indeed a wide spectrum of different risk profiles: for women with high-risk disease, if chemotherapy is appropriate, others will derive little benefit from it. Our study therefore represents a significant advance for this population of patients, which consists of two third of all breast cancers. We have indeed demonstrated that the CycHyp signature outperforms the existing major prognostic gene expression signatures and offers a unique decision making tool to complement the discrimination of breast cancer patients based on anatomopathologic evaluation.

More generally, the excellent prognostic value of CycHyp confirms the link between cycling hypoxia and cancer aggressiveness [4, 5]. This gives credentials to the phenotypic adaptation of tumors resulting from heterogeneities in blood flow distribution as a trigger of cancer progression [3, 4]. Also, with the recent impetus in the understanding of tumor metabolism [37, 38], it has become obvious that the capacity of a given tumor cell to survive in both aerobic and anaerobic environments represents a critical advantage [39-41]. Interestingly, our study also documents the higher prognostic value of a transcriptomic signature derived from cycling hypoxia *vs.* continuous hypoxia. This confirms that although hypoxia is a frequent feature of poor-prognosis tumors and was reported to drive gene signature associated with negative outcome [42-45], prognostic markers integrating fluctuations in the hypoxic status of tumors (this study) introduce an additional layer of complexity that better fits the *in vivo* situation.

Whether the CycHyp signature encompasses genes that actively drive cancer progression or reflects a context of metabolic and hypoxic stress favorable to increased mutagenesis and genetic instability [3], warrants further studies. A few hints can however be gleaned from the comparison of the different signatures.

First, the comparison of the CycHyp and ContHyp signatures indicates that the cycling nature of hypoxia leads to specific alterations in mRNA expression since only 11 common transcripts were found in the two gene lists (see symbols # in Table 1). Furthermore, among these 11 genes, most encode for proteins involved in housekeeping functions such as chromatin packaging (HIST1H 1C, 2AC, 4A and 4C) and RNA processing (RPS13 and 28). The only gene common to the two signatures with a known function related to hypoxia is RBX1 or E3 ubiquitin ligase which mediates the ubiquitination and subsequent proteasomal degradation of target proteins [46], including the misfolded proteins known to accumulate under low pO_2_. Besides the RBX1 gene, the CycHyp signature does not actually contain genes known to be consistently regulated in response to chronic hypoxia. By contrast, the ContHyp signature contains 14 genes already reported to be overexpressed under low pO_2_ and even directly under the control of the transcription factor HIF-1α, including those coding for glucose metabolism enzymes (ALDOA, PFKB3, PFKB4, PGK1, PGAM1, GPI) and the angiogenic growth factor VEGFA. This HIF-dependent gene expression program of the ContHyp signature was actually confirmed in the GSEA and MSigBD analyses and was consistent with previously reported hypoxia-driven gene signatures [42, 44, 45]. More generally, these findings position the CycHyp signature far from the conventional hypoxia-derived signatures [29, 30] but instead as a biomarker of a distinct tumor biology process involving adaptation to fluctuations in the tumor microenvironment.

Second, a large amount of transcripts of the CycHyp signature encode for proteins themselves involved in the regulation of transcription. Data mining revealed that more than 18 transcripts of the CycHyp signature are transcription factors/regulators and 13 others are directly involved in RNA processing (see symbols * and § in Table 1, respectively). This represents one third of the genes comprising the CycHyp signature and reflects a major difference with the ContHyp signature. While hypoxia is usually associated with cell cycle arrest and mTOR inhibition, cycling hypoxia may be compatible with a maintained proliferation potential. This is further supported by the suppression of geroconversion (ie, the process leading from proliferative arrest to irreversible senescence) observed in response to hypoxia [47, 48] that offers tumor cells the opportunity to re-enter cell cycle when O_2_ is again available. Further studies are needed to compare the evolution of mTOR activity and mTOR-dependent genes (including those encoding for ribosomal proteins) during cycling and continuous hypoxia.

Finally, the *in vitro* conditions at the origin of the establishment of the CycHyp signature may actually have specific bearing on its robustness and applicability. Indeed, we previously documented that fluctuating oxygen levels could also directly impact endothelial cells within a tumor [49, 50] indicating that non-tumor cells may also contribute to the same transcriptomic adaptation as tumor cells, thereby reinforcing the relevance of the CycHyp signature. Also, although we have used the CycHyp signature as a prognostic biomarker for early-stage breast cancer, this signature was identified by integrating the information arising from tumor cells of various origins and characterized by various oncogenic alterations; the prognostic value of the CycHyp signature in other cancers is currently under investigation in our laboratory.

Altogether, the above findings indicate that the CycHyp signature represents a new generation of prognostic biomarker reflecting a generic environmental condition in tumors that differs from the conventional view of a static, continuous hypoxia occurring in tumors. When applied to breast cancer, the CycHyp signature has a powerful prognostic value independently of molecular risk factors but also offers a unique decision making tool to complement the discrimination of patients based on anatomopathologic evaluation. The CycHyp signature is distinct from conventional hypoxia-related gene signature but also from existing prognostic metagenes, and the rationale behind its discovery supports a potential broad applicability to evaluate cancer patient outcomes.

## MATERIALS AND METHODS

### Tumor cells

Twenty cell lines derived from cancer patients (see Suppl. Table 1 for details) were submitted to cycling hypoxia (CycHyp), i.e. 24 cycles of 30 min incubation under normoxia and 30 min incubation under hypoxic (1% O_2_) conditions to reproduce tumor hypoxic fluctuations, as previously reported [5, 51]. We also considered control conditions of 24 h continuous exposure of tumor cells to either 21% O_2_ (Normoxia) or 1% O_2_ (ContHyp). For each culture condition, cells were immediately snap-frozen at the end of the last incubation period.

### Identification of the signatures

mRNA extracts from each tumor cell cultured under the three above conditions (normoxia, cycling hypoxia and continuous hypoxia) were analysed by hybridization on Human Gene 1.0 ST Affymetrix microarrays (GEO access number: GSE42416):

http://www.ncbi.nlm.nih.gov/geo/query/acc.cgi?token=probzowmiyseqxm&acc=GSE42416

The extent of the resulting tumor cell datasets (20 samples in each of the three conditions) led us to resort on a resampling mechanism to increase the robustness of the signatures to be identified. For every resampling experiment, a subset of 90 % of the samples was chosen uniformly at random as a training set and the remaining 10% were used as validation set. Differentially expressed probesets (one probeset = a collection of probes designed to interrogate a given sequence) were assessed on each subset according to a t-test and the corresponding FDR corrected p-values were reported. The 100 probesets with the lowest corrected p-values, averaged over 200 resamplings [52-54], formed the CycHyp (Table 1) or ContHyp (Table 2) signatures. All such expression differences were highly significant (p<1e-4) after Benjamini-Hochberg FDR correction for the multiplicity of the test [55]. Of note, in each resampling, the 10 % data not used to select probesets allowed one to estimate the discrimination potential between (cycling or continuous) hypoxia versus normoxia conditions. The average classification accuracy over all resamplings amounted to 97.5 % for CycHyp and 94.3% for ContHyp.

The 100 HGU1.0 ST probesets forming the CycHyp signature corresponded to 94 unique Entrez GeneID in the NCBI database, out of which 69 genes were available on the HGU133a platform (i.e., the technology used in most clinical studies considered here). Those 69 genes were represented by 87 HGU133a probesets. The few datasets collected on HGU133plus2 were reduced to the probesets also present on HGU133a.

### Patient data sets

All breast cancer expression data were summarized with MAS5 and represented in log2 scale (except for GSE6532 already summarized with RMA). Breast cancer subtypes (ER+/HER2-, ER-/HER2- and HER2+) were identified with the genefu R package [56] (see Supplementary R Package). Disease-free survival at 5 years was used as the survival endpoint. The data from all patients were censored at 10 years to have comparable follow-up times across clinical studies [32].

### Prognostic models of the clinical outcome

The VDX dataset (GSE2034 and GSE5327 from the GEO database) was considered as a reference because of its large number of node-negative untreated patients [17]. This dataset formed the training set used to estimate a prognostic model of the clinical outcome. A risk score for each patient was computed from a penalized Cox proportional hazards model [57] implemented in the Penalized R package [58]; the parameters of the elastic net penalty were learned on the training set by cross-validation. Prediction into a high risk *vs.* low risk group resulted from a predefined threshold value on this risk score. The decision threshold was chosen on the training set to maximize the specificity and sensitivity of the discrimination between patients with progressing disease versus disease-free patients at 5 years. Following the methodology described by Haibe-Kains et al. [32], all other datasets were used as validations to assess the prognostic performances on independent samples, i.e. balanced classification rate (BCR), concordance index (CI) [59] and hazard ratio (HR) [60]. The survcomp R packages were used to test the significance of the HR and CI values [33] while a Z-test allowed to infer p-values for the BCR relying on an approximation by a normal distribution.

Prognostic performances of a penalized Cox model defined on the CycHyp signature were also compared with well-established prognosis models for breast cancer, namely Gene 70 (Mammaprint) [14], Gene 76 [17] and Oncotype DX [15] signatures. Those existing signatures were associated to specific prognostic models implemented in the genefu R package [56]. Comparison of CycHyp and ContHyp signatures was also carried out with random gene signatures of the same sizes, i.e. 87 and 123 probesets, respectively. One thousand signatures of each size were generated and analysed using the methodology described by Venet et al. [11]. The objective of those experiments was to assess to which extent the CycHyp and ContHyp signatures had a better discrimination power between risk groups than random signatures. Gene Set Enrichment Assay (GSEA) analysis was also performed using the molecular signature database (MSigDB) and the CycHyp and ContHyp signatures expanded to 2118 and 2065 differentially expressed genes, respectively (after FDR correction and averaged over all resamplings.

## SUPPLEMENTARY MATERIAL TABLES AND FIGURES


