# Supplementary material for: A generic cycling hypoxia-derived prognostic gene signature: application to breast cancer profiling
Source: Oncotarget. 2014 Jul 31;5(16):6947–63. doi: 10.18632/oncotarget.2285 (PMC4196175; doi:10.18632/oncotarget.2285)
Supplement: Supplementary file 1 [file oncotarget-05-6947-s001.pdf]

A generic cycling hypoxia-derived prognostic gene signature: application to breast cancer profiling

Supplementary Material

Supplementary Table 1: List of Human Tumor Cells used for Microarray Analysis.

Cell line	Organ	Disease
MCF-7	Breast	Adenocarcinoma
MDA-MB-231	Breast	Adenocarcinoma
T47D	Breast	Ductal carcinoma
A549	Lung	Carcinoma
Widr	Colon	Colorectal adenocarcinoma
HCT116 ^{WTP53}	Colon	Colorectal carcinoma
HCT116 $^{-/-P53}$	Colon	Colorectal carcinoma
HT29	Colon	Colorectal adenocarcinoma
Colo-205	Colon	Colorectal adenocarcinoma
LoVo	Colon	Colorectal adenocarcinoma
HCT15	Colon	Colorectal adenocarcinoma
SiHa	Cervix	Squamous cell carcinoma
PC3	Prostate	Adenocarcinoma
U373	Brain	Glioblastoma
HepG2	Liver	Hepatocellular carcinoma
Hep3B	Liver	Hepatocellular carcinoma
PLC/PRF/5	Liver	Hepatoma
SK-HEP-1	Liver	Adenocarcinoma
A498	Kidney	Carcinoma
HT1080	Connective tissue	Fibrosarcoma

Cancer cells were acquired from the ATCC where they are regularly authenticated. Cells were stored according to the supplier's instructions and used within 6 months after resuscitation of frozen aliquots.

Supplementary Table 2: GSEA analysis on the CycHyp signature extented to 2,118 differentially expressed genes between normoxia and cycling hypoxia. The table contains the signature sizes, primary and normalized enrichment scores (ES and NES, respectively), the nominal p-values, the false discovery rates (FDR) q-values and the familywise-error rate (FWER) for the top 50 gene sets enriched in the CycHyp signature. Only the two first gene sets are significantly enriched with a FDR q-value < 0.1.

Gene sets	Size	ES	NES	NOM p---val	FDR q---val	FWER p---val
JISON SICKLE CELL DISEASE DN	26	0,442	2,653	0	0,011	0,012
ZWANG TRANSIENTLY UP BY 2ND EGF PULSE ONLY	226	0,158	2,524	0	0,016	0,036
REACTOME TRANSLATION	40	0,275	1,997	0,006	0,44	0,771
PECE MAMMARY STEM CELL DN	29	0,303	1,926	0,008	0,501	0,894
KRIGE RESPONSE TO TOSEDOSTAT 24HR DN	94	0,12	1,312	0,155	0,978	1
BURTON ADIPOGENESIS 5	15	0,282	1,296	0,168	0,986	1
HAMAI APOPTOSIS VIA TRAIL DN	17	0,269	1,322	0,151	0,99	1
HSIAO HOUSEKEEPING GENES	65	0,143	1,336	0,14	0,99	1
MULLIGHAN MLL SIGNATURE 2 DN	15	0,277	1,27	0,175	0,992	1
REACTOME RNA POL I TRANSCRIPTION	17	0,267	1,315	0,16	0,992	1
HUTTMANN B CLL POOR SURVIVAL UP	20	0,26	1,383	0,12	0,993	1
REACTOME PEPTIDE CHAIN ELONGATION	27	0,202	1,241	0,206	0,994	1
WANG TUMOR INVASIVENESS UP	46	0,182	1,437	0,094	0,999	1
BOUDOUKHA BOUND BY IGF2BP2	15	0,371	1,74	0,024	1	0,998
BURTON ADIPOGENESIS 6	16	0,304	1,44	0,094	1	1
LI INDUCED T TO NATURAL KILLER UP	15	0,301	1,399	0,111	1	1
KEGG UBIQUITIN MEDIATED PROTEOLYSIS	22	0,29	1,608	0,045	1	1
KEGG SYSTEMIC LUPUS ERYTHEMATOSUS	16	0,289	1,387	0,118	1	1
GOLDRATH ANTIGEN RESPONSE	19	0,286	1,488	0,078	1	1
WIERENGA STAT5A TARGETS DN	18	0,285	1,45	0,082	1	1
YAO TEMPORAL RESPONSE TO PROGESTERONE CLUSTER 17	21	0,281	1,526	0,064	1	1
LINDGREN BLADDER CANCER CLUSTER 3 UP	16	0,271	1,297	0,163	1	1
MALONEY RESPONSE TO 17AAG DN	18	0,264	1,346	0,144	1	1
UDAYAKUMAR MED1 TARGETS UP	19	0,26	1,34	0,145	1	1
AFFAR YY1 TARGETS UP	20	0,259	1,357	0,137	1	1
KEGG PATHWAYS IN CANCER	28	0,252	1,563	0,054	1	1
SWEET LUNG CANCER KRAS DN	26	0,25	1,492	0,076	1	1
REACTOME 3 UTR MEDIATED TRANSLATIONAL REGULATION	32	0,249	1,638	0,033	1	1
YAGI AML WITH T 8 21 TRANSLOCATION	20	0,249	1,325	0,147	1	1
KEGG WNT SIGNALING PATHWAY	19	0,239	1,241	0,208	1	1
GARY CD5 TARGETS UP	31	0,228	1,482	0,073	1	1
REACTOME SRP DEPENDENT COTRANSLATIONAL PROTEIN TARGETING TO MEMBRANE	33	0,227	1,514	0,064	1	1
NIKOLSKY BREAST CANCER 16P13 AMPLICON	26	0,227	1,364	0,118	1	1
MOHANKUMAR TLX1 TARGETS UP	27	0,224	1,372	0,122	1	1
NIKOLSKY BREAST CANCER 17Q21 Q25 AMPLICON	36	0,217	1,508	0,068	1	1
WONG EMBRYONIC STEM CELL CORE	46	0,196	1,527	0,062	1	1
YAGI AML WITH INV 16 TRANSLOCATION	34	0,186	1,255	0,197	1	1
MIKKELSEN ES ICP WITH H3K4ME3	54	0,175	1,473	0,076	1	1
KRIGE RESPONSE TO TOSEDOSTAT 6HR UP	42	0,167	1,254	0,192	1	1
STARK PREFRONTAL CORTEX 22Q11 DELETION DN	71	0,166	1,586	0,047	1	1
DELACROIX RAR BOUND ES	45	0,164	1,274	0,178	1	1
MOOTHA MITOCHONDRIA	65	0,152	1,416	0,099	1	1
MEISSNER BRAIN HCP WITH H3K27ME3	55	0,151	1,28	0,176	1	1
MARTENS TRETINOIN RESPONSE DN	108	0,148	1,722	0,028	1	0,999
KRIGE RESPONSE TO TOSEDOSTAT 6HR DN	87	0,141	1,485	0,073	1	1
REACTOME METABOLISM OF PROTEINS	75	0,141	1,385	0,113	1	1
MIKKELSEN MEF HCP WITH H3K27ME3	89	0,137	1,454	0,085	1	1
ACEVEDO LIVER TUMOR VS NORMAL ADJACENT TISSUE UP	94	0,129	1,406	0,103	1	1
BENPORATH EED TARGETS	115	0,104	1,249	0,196	1	1
DANG BOUND BY MYC	127	0,102	1,274	0,174	1	1

Supplementary Table 3: GSEA analysis on the ContHyp signature extended to 2,065 differentially expressed genes between normoxia and continuous hypoxia. The table contains the signature sizes, primary and normalized enrichment scores (ES and NES, respectively), the nominal p-values, the false discovery rates (FDR) q-values and the familywise-error rate (FWER). 17 gene sets (red) were previoulsy identified as related to hypoxia.

Gene sets	Size	ES	NES	NOM p---val	FDR q---val	FWER p---val
FARDIN HYPOXIA 11	17	0,712	3,492	0	0	0
LEONARD HYPOXIA	21	0,524	2,841	0	0	0,002
MENSE HYPOXIA UP	39	0,477	3,469	0	0	0
ELVIDGE HYPOXIA BY DMOG UP	46	0,459	3,623	0	0	0
ELVIDGE HIF1A AND HIF2A TARGETS DN	44	0,445	3,376	0	0	0
WINTER HYPOXIA METAGENE	46	0,443	3,444	0	0	0
QI HYPOXIA	36	0,421	2,943	0	0	0
ELVIDGE HIF1A TARGETS DN	43	0,39	2,959	0	0	0
ELVIDGE HYPOXIA UP	56	0,386	3,326	0	0	0
KRIEG HYPOXIA NOT VIA KDM3A	113	0,295	3,474	0	0	0
NAKAMURA TUMOR ZONE PERIPHERAL VS CENTRAL DN	76	0,27	2,641	0	0,002	0,015
PRAMOONJAGO SOX4 TARGETS UP	15	0,551	2,582	0	0,003	0,029
WINTER HYPOXIA UP	20	0,491	2,596	0	0,003	0,024
GROSS HYPOXIA VIA HIF1A DN	23	0,432	2,457	0	0,007	0,083
GROSS HYPOXIA VIA ELK3 AND HIF1A UP	29	0,392	2,466	0	0,007	0,079
BASAKI YBX1 TARGETS DN	35	0,359	2,479	0	0,007	0,07
MARTORIATI MDM4 TARGETS NEUROEPITHELIUM UP	23	0,432	2,448	0	0,008	0,091
MARKEY RB1 ACUTE LOF UP	24	0,419	2,432	0	0,008	0,103
SCHLOSSER MYC TARGETS AND SERUM RESPONSE DN	18	0,48	2,412	0	0,009	0,117
MANALO HYPOXIA UP	35	0,35	2,419	0	0,009	0,113
HARRIS HYPOXIA	17	0,488	2,383	0,002	0,01	0,143
REACTOME METABOLISM OF RNA	60	0,264	2,351	0,001	0,012	0,177
BOYAULT LIVER CANCER SUBCLASS G3 UP	44	0,301	2,318	0,001	0,014	0,22
BLALOCK ALZHEIMERS DISEASE DN	169	0,163	2,328	0	0,014	0,208
DANG BOUND BY MYC	170	0,162	2,296	0,002	0,016	0,253
PID HIF1 TFPATHWAY	16	0,477	2,277	0,002	0,017	0,283
DANG MYC TARGETS UP	44	0,3	2,281	0,001	0,017	0,278
SHAFFER IRF4 TARGETS IN MYELOMA VS MATURE B LYMPHO	15	0,488	2,263	0	0,018	0,304
WEI MYCN TARGETS WITH E BOX	194	0,149	2,226	0,001	0,022	0,372
KRIGE RESPONSE TO TOSEDOSTAT 24HR DN	220	0,139	2,21	0,001	0,024	0,408
REACTOME INFLUENZA LIFE CYCLE	33	0,322	2,173	0,002	0,03	0,492
NUYTTEN EZH2 TARGETS UP	78	0,217	2,169	0,002	0,03	0,501
GROSS HYPOXIA VIA ELK3 DN	29	0,339	2,156	0,002	0,031	0,535
BENPORATH MYC MAX TARGETS	120	0,176	2,146	0,001	0,032	0,558
CAIRO HEPATOBLASTOMA CLASSES UP	139	0,167	2,158	0,003	0,032	0,53
PODAR RESPONSE TO ADAPHOSTIN UP	25	0,352	2,077	0,003	0,049	0,725
KIM MYC AMPLIFICATION TARGETS UP	33	0,306	2,062	0,004	0,053	0,762
YAMAZAKI TCEB3 TARGETS DN	25	0,349	2,05	0,005	0,055	0,786
RODRIGUES THYROID CARCINOMA POORLY DIFFERENTIATED	113	0,168	2,02	0,005	0,064	0,845
REACTOME RNA POL I PROMOTER OPENING	16	0,415	2,004	0,006	0,069	0,87
HSIAO HOUSEKEEPING GENES	72	0,208	1,997	0,004	0,071	0,88
GARY CD5 TARGETS DN	109	0,17	1,98	0,005	0,073	0,903
DODD NASOPHARYNGEAL CARCINOMA DN	233	0,122	1,983	0,002	0,074	0,9
GRAESSMANN APOPTOSIS BY SERUM DEPRIVATION DN	28	0,322	1,984	0,006	0,075	0,899
YAO TEMPORAL RESPONSE TO PROGESTERONE CLUSTER 11	31	0,301	1,965	0,006	0,078	0,924
KRIGE RESPONSE TO TOSEDOSTAT 6HR DN	188	0,131	1,947	0,007	0,083	0,941
GRAESSMANN RESPONSE TO MC AND DOXORUBICIN DN	123	0,158	1,949	0,004	0,084	0,94
LEE BMP2 TARGETS DN	214	0,125	1,942	0,008	0,084	0,946
REACTOME RNA POL I TRANSCRIPTION	20	0,363	1,936	0,006	0,085	0,951
WANG CISPLATIN RESPONSE AND XPC DN	24	0,329	1,928	0,007	0,087	0,96
LOCKWOOD AMPLIFIED IN LUNG CANCER	27	0,312	1,905	0,009	0,098	0,975
PECE MAMMARY STEM CELL DN	29	0,297	1,899	0,011	0,099	0,978

Supplementary Table 4: Overlap, in terms of number of common genes, between the CycHyp signature and gene sets from the MsigDB identified as being related to hypoxia or HIF.

	Hypoxia Gene Set	Gene in common
1	PID HIF1APATHWAY	1
2	REACTOME REGULATION OF HYPOXIA INDUCIBLE FACTOR HIF BY OXYGEN	2
3	GROSS HYPOXIA VIA ELK3 AND HIF1A DN	1
4	REACTOME OXYGEN DEPENDENT PROLINE HYDROXYLATION OF HYPOXIA INDUCIBLE FACTOR ALPHA	2
5	WINTER HYPOXIA UP	1
6	WINTER HYPOXIA DN	1
7	ELVIDGE HYPOXIA BY DMOG DN	1
8	GROSS HYPOXIA VIA ELK3 UP GROSS	2
9	HYPOXIA VIA ELK3 ONLY DN	1
10	MANALO HYPOXIA DN	1
11	JIANG HYPOXIA NORMAL	1
12	JIANG HYPOXIA CANCER	1
13	KRIEG HYPOXIA NOT VIA KDM3A	4

Supplementary Table 5: Overlap, in terms of number of common genes, between the ContHyp signature and gene sets from the MsigDB identified as being related to hypoxia or HIF.

	Hypoxia Gene Set	Gene in common
1	BIOCARTA HIF PATHWAY	1
2	PID HIF2PATHWAY	2
3	PID HIF1APATHWAY	1
4	PID HIF1 TFPATHWAY	5
5	REACTOME REGULATION OF HYPOXIA INDUCIBLE FACTOR HIF BY OXYGEN	2
6	ELVIDGE HIF1A TARGETS UP	2
7	ELVIDGE HIF1A TARGETS DN	15
8	ELVIDGE HIF1A AND HIF2A TARGETS DN	16
9	GROSS HYPOXIA VIA HIF1A ONLY	1
10	GROSS HIF1A TARGETS DN	3
11	GROSS HYPOXIA VIA HIF1A DN	4
12	GROSS HYPOXIA VIA ELK3 AND HIF1A UP	14
13	RANKIN ANGIOGENIC TARGETS OF VHL HIF2A DN	1
14	SEMENZA HIF1 TARGETS	4
15	QI HYPOXIA TARGETS OF HIF1A AND FOXA2	1
16	REACTOME OXYGEN DEPENDENT PROLINE HYDROXYLATION OF HYPOXIA INDUCIBLE FACTOR ALPHA	1
17	WINTER HYPOXIA UP	11
18	ELVIDGE HYPOXIA UP	19
19	ELVIDGE HYPOXIA DN	4
20	ELVIDGE HYPOXIA BY DMOG UP ELVIDGE	17
21	HYPOXIA BY DMOG DN WEINMANN	2
22	ADAPTATION TO HYPOXIA UP WEINMANN	1
23	ADAPTATION TO HYPOXIA DN KONDO	1
24	HYPOXIA	1
25	GROSS HYPOXIA VIA ELK3 UP	4
26	GROSS HYPOXIA VIA ELK3 DN	6
27	GROSS HYPOXIA VIA ELK3 ONLY UP	1
28	MANALO HYPOXIA DN	5
29	MANALO HYPOXIA UP	10
30	MENSE HYPOXIA UP	19
31	KIM HYPOXIA	4
32	HARRIS HYPOXIA	7
33	LEONARD HYPOXIA	12
34	JIANG HYPOXIA NORMAL	9
35	JIANG HYPOXIA CANCER	2
36	JIANG AGING HYPOTHALAMUS UP	1
37	WINTER HYPOXIA METAGENE	16
38	MIZUKAMI HYPOXIA UP	1
39	QI HYPOXIA FARDIN	14
40	HYPOXIA 9	5
41	FARDIN HYPOXIA 11	14
42	WACKER HYPOXIA TARGETS OF VHL	3
43	KRIEG HYPOXIA VIA KDM3A	1
44	KRIEG HYPOXIA NOT VIA KDM3A	27

Supplementary Table 6: Overlap, in terms of number of common genes, between the CycHyp or ContHyp signatures and the conventional hypoxia-related signatures (respective sizes in the second column) described by Seigneuric et al. (2007) and Starmans et al. (2012).

		Size	CycHyp	ContHyp
Seigneuric et al. (2007)	Early 0%	72	0	0
	Late 0%	71	1	7
	Early 2%	34	1	0
	Late 2%	32	0	3
Starmans et al. (2012)	Cluster 1	69	0	5
	Cluster 2	246	1	20
	Cluster 3	157	0	4
	Cluster 4	95	1	1
	Cluster 5	162	0	0
	Cluster 6	14	0	0
	Cluster 7	28	1	0
	Upregulated	780	2	32
	Downregulated	656	6	6

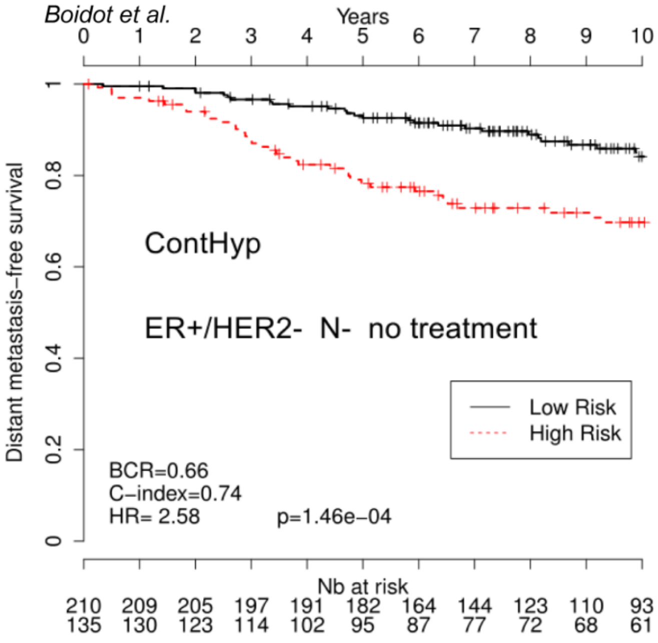

Supplementary Figure 1: Kaplan-Meier survival curves of node-negative, untreated ER+/HER2- patients as determined by using the ContHyp signature (DFS Mantel-Cox comparison).

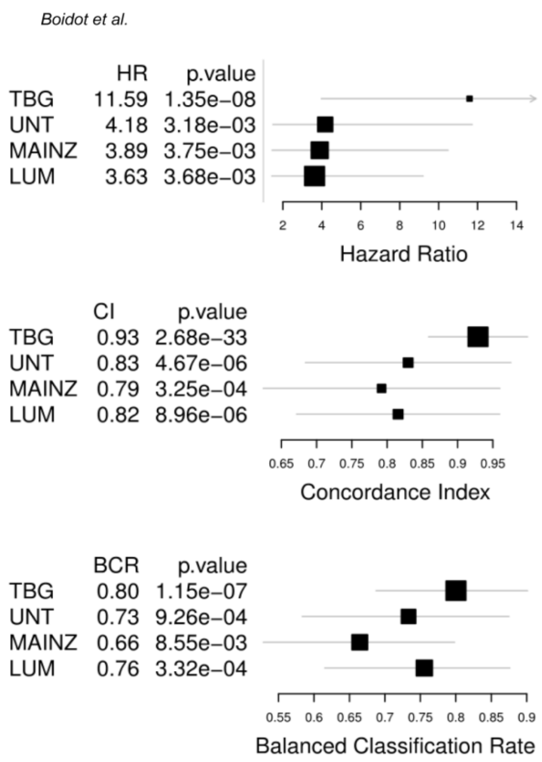

Supplementary Figure 2: Forest plots of the hazard ratio (HR), Concordance index (CI) and balance classification rate (BCR) for the prediction in high risk vs. low risk groups for each of the 4 data sets that form the total collection of node-negative, untreated ER+/HER2- patients; data are presented with their associated p-values.

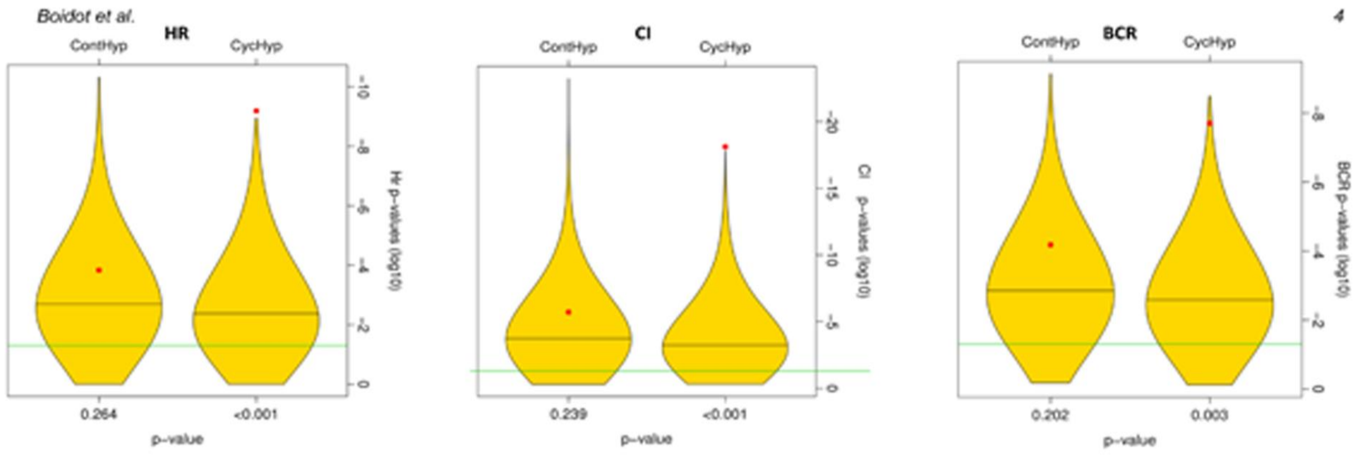

Supplementary Figure 3: Graphs represent the hazard ratio (HR), concordance index (CI) and balanced classification rate (BCR) (expressed as the logarithm of the corresponding p-values) related to the power of discrimination in high vs. low risk groups, of the ContHyp and CycHyp signatures (see red dots) versus 1,000 randomly generated signatures (yellow shapes depicting their distribution).

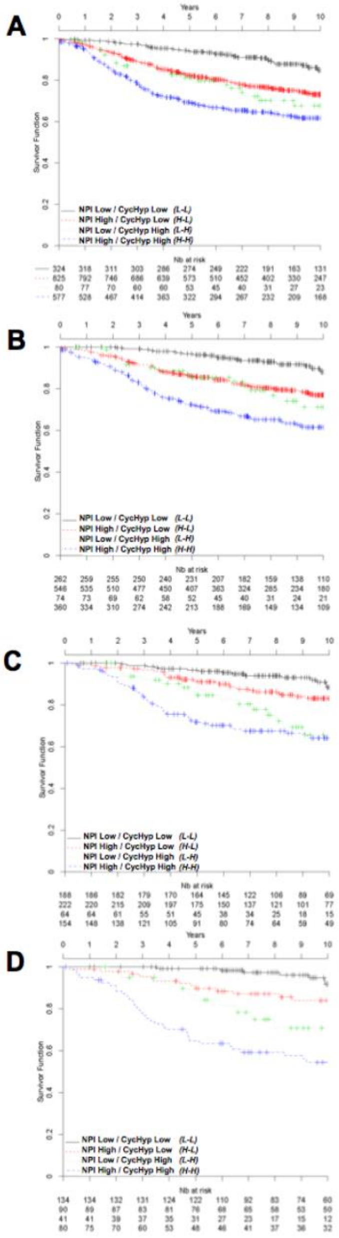

All patients

		H-L	L-H	H-H
L-L	HR	**2.33**	**2.96**	**3.86**
	p	**3.73e-07**	**2.20e-04**	**1.49e-17**
H-L	HR		1.22	**1.69**
	p		0.392	**3.04e-07**
L-H	HR			1.39
	p			0.135

ER+ HER2- (N+/N-)

		H-L	L-H	H-H
L-L	HR	**2.72**	**3.35**	**5.32**
	p	**3.45e-06**	**5.67e-04**	**7.28e-17**
H-L	HR		1.17	**1.97**
	p		0.569	**3.63e-07**
L-H	HR			1.70
	p			0.034

ER+ HER2- (N-)

		H-L	L-H	H-H
L-L	HR	**2.11**	**4.41**	**5.61**
	p	**1.89e-02**	**9.83e-05**	**4.38e-10**
H-L	HR		**2.03**	**2.72**
	p		**2.82e-02**	**9.12e-06**
L-H	HR			1.37
	p			0.265

ER+ HER2- (N-) untreated

		H-L	L-H	H-H
L-L	HR	**3.93**	**7.81**	**14.34**
	p	**5.42e-03**	**1.11e-04**	**9.17e-13**
H-L	HR		1.88	**3.63**
	p		1.43e-01	**2.48e-05**
L-H	HR			1.98
	p			0.044

Supplementary Figure 4: Kaplan-Meier survival curves of patients with primary breast cancer stratified at low or high risk according to the CycHyp signature and the NPI nomenclature (DFS Mantel-Cox comparison). (A) All patients, (B.) ER+/HER2- patients, (C.) node-negative ER+/HER2, (D.) node-negative, untreated ER+/HER2- patients. Indicated p-values are derived from Mantel- Cox log-rank tests.
